# Helical 4D CT pitch management for the Brilliance CT Big Bore in clinical practice

**DOI:** 10.1120/jacmp.v16i3.5111

**Published:** 2015-05-08

**Authors:** Guido Hilgers, Tonnis Nuver, André Minken

**Affiliations:** ^1^ Department of Medical Physics Radiotherapiegroep | Deventer Deventer The Netherlands

**Keywords:** 4D CT, pitch, motion artifacts

## Abstract

In external beam radiotherapy treatment planning for patients with thoracic malignancies, respiratory‐correlated CT (4D CT) is used to obtain high quality studies in the presence of respiratory motion. When helical 4D CT scans are acquired with a Brilliance CT Big Bore, the pitch must meet two conditions. It must be low enough to avoid motion artifacts, and high enough to cover the entire scan length within 120 s to prevent overheating of the X‐ray tube. We developed a nomogram that can be used to obtain a suitable pitch satisfying both requirements. We also assessed the effects on the image quality of a pitch that exceeds the maximum pitch, and of a field of view (FOV) reduction. It was shown that, for AV G and MIP reconstructions, the manufacturer's maximum pitch equation yields an underestimation due to its FOV term.

PACS number: 87.57.Q‐, 87.57.cp

## I. INTRODUCTION

In external beam radiotherapy treatment planning for patients with thoracic malignancies, respiratory‐correlated CT (4D CT) is used to obtain high quality studies in the presence of respiratory motion.^(1)^ When 4D CT scans are acquired in helical mode, the pitch — defined as the table travel per gantry rotation divided by the detector collimation — must be low enough to illuminate each voxel of patient anatomy for an entire respiratory cycle.^(2)^ This requirement can be translated into a maximum pitch that depends on the patient's respiratory frequency. When the actual pitch exceeds the maximum pitch, motion artifacts are expected to occur.

The Brilliance CT Big Bore (Philips Healthcare, Cleveland, OH) is capable of performing helical 4D CT scans. However, its design puts an additional requirement on the pitch. Since the manufacturer has limited the maximum scan time to 120 s, to prevent overheating of the X‐ray tube,^(3)^ the pitch must be high enough to make sure that the entire scan length can be covered during this timeframe. This requirement can be translated into a minimum pitch that depends on the scan length and, therefore, on the patient's anatomy and tumor location(s). If the actual pitch is lower than the minimum pitch, the planning study is shorter than desired and might not be suited for treatment planning.

In this paper, we will present a nomogram that can be used to obtain a pitch satisfying both requirements. We will also assess the effects on the image quality of a pitch that exceeds the maximum pitch, and of a field of view reduction.

## II. MATERIALS AND METHODS

### A. Nomogram

To construct the nomogram, respiratory frequencies were linked to maximum pitches and scan lengths. The maximum pitch ($p_{\text{max}}$) is given by the manufacturer in a white paper:^(3)^

(1) $$p_{\max}=\left(\frac{t_{rot}\cdot f}{60}\right)\left(1-\frac{FOV}{2R}\right)$$

where $t_{\mathrm{rot}}$ is the gantry rotation time (in s), *f* is the patient's respiratory frequency (in breaths per minute), *FOV* is the size of the reconstructed field of view (in mm), and *R* is the distance from the focus to the CT isocenter (645 mm for a Brilliance CT Big Bore). The first term originates from the work of Keall et al.^(2)^ and gives the maximum pitch in the CT isocenter. The second term ensures that also voxels at the edge of the reconstructed FOV are illuminated for an entire respiratory cycle.^(3)^

The maximum scan length ($L_{\text{max}}$, in mm) at a certain pitch *p* can be calculated from the table speed and the maximum scan time of 120 s. The table speed (v, in $\text{mm}\cdot L^{-1}$) is:^(4)^

(2) $$v=\frac{p\cdot (N\times T)}{t_{rot}}$$

where N×T is the detector collimation (in mm). The maximum scan length ($L_{\text{max}}$, in mm) can then be calculated as follows:

(3) $$L_{\max}=120v-\Delta L$$

where ΔL is the difference between exposed and planned length. This difference, which is called overranging and is inherent to helical CT, is described in detail by Schilham et al.^(5)^

Substituting *v* in Eq. (3) with Eq. (2) yields:

(4) $$L_{\max}=\frac{120p\cdot (N\times T)}{t_{rot}}-\Delta L$$

In order to maximize the maximum scan length, *p* needs to be maximized. This is achieved by setting p to $p_{\text{max}}$ using Eq. (1):

(5) $$L_{\max}=2f(N\times T)\left(1-\frac{FOV}{2R}\right)-\Delta L$$

The values of two acquisition parameters must be known to calculate the maximum pitch and the maximum scan length. The maximum pitch depends on the selected gantry rotation time, whereas the maximum scan length depends on the selected detector collimation. When a 4D CT scan protocol is created using the “PULMO GATING” scan type, which is advised by the manufacturer, two different gantry rotation times (0.44 and 0.5 s) and two different detector collimations (16×0.075 and 16×1.5 mm) are available.

With Eq. (1), respiratory frequencies of 8 to 26 breaths per minute (BPM) were linked to maximum pitches for both gantry rotation times and a typical FOV of 500 mm. Before Eq. (5) was used to link these respiratory frequencies to maximum scan lengths, the unknown overranging (ΔL) was experimentally determined.

The scanner software (version 3.5.4) shows a notification when the scan length is at its maximum and cannot be increased any further. This feature was used to determine ΔL using Eq. (4) for each of the four combinations of gantry rotation time and detector collimation with pitches of approximately 0.04, 0.06, 0.08, 0.10, 0.12, 0.14, and 0.16.

First, a scanogram was made. Then, the gantry rotation time, detector collimation, and the pitch were set. Subsequently, for a primary slice width of 2 mm, the scan length was increased until the notification appeared. This scan length was recorded as $L_{\text{max}}$ and ΔL was calculated. Finally, Eq. (5) was used to link the respiratory frequencies of 8 to 26 BPM to maximum scan lengths for both detector collimations. Again, a typical FOV of 500 mm was used.

### B. Phantom studies

#### B.1 Effect $p>p_{\text{max}}$

**Figure 1:**
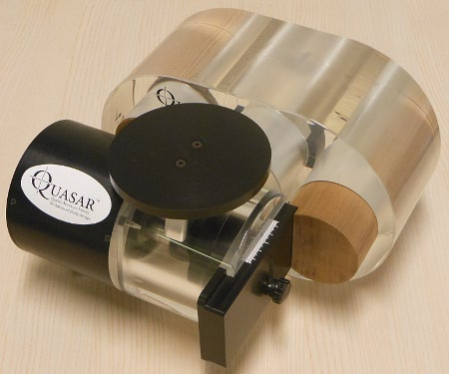
QUASAR respiratory motion phantom.

To assess the effect of $p>p_{\text{max}}$, we performed a phantom study. A QUASAR respiratory motion phantom (Modus Medical Inc., London, ON) was used to simulate a breathing patient. The phantom (see Fig. 1) consists of an acrylic “thorax” (L=120 mm, w=300 mm,h=200 mm) with two cylindrical cedar “lungs”. One of the cylinders is connected to a driving mechanism. In this cylinder (L=180 mm, d=80 mm), a polystyrene sphere (r=15 mm) is embedded that represents a tumor. The driving mechanism moves the cylinder back and forth in a sinusoidal pattern of the form:

(6) $$z(t)=a\sin\left(\frac{2\pi t}{T}\right)$$

where z(t) is the position of the center of the sphere (in mm) along the superior–inferior axis at time *t* (in s), *a* is the amplitude (in mm), and *T* is the oscillation period (in s). The driving mechanism is also connected to a platform, which moves up and down to represent chest wall movement. The oscillation periods of the chest wall platform and the cylinder are equal. The amplitude of the chest wall platform is fixed, whereas the amplitude of the cylinder is adjustable.

Seven scans $\left(t_{\text{rot}}=0.5\;s,\;N\times T=16\times 1.5\;\text{mm},\;120\;\text{kVp},\;800\;\text{mAs}/\text{slice}\right)$ were made of the phantom with a Brilliance CT Big Bore running software version 3.5.4. The first scan was made with a=0 mm (simulation of a stationary tumor), whereas the other scans were made with a=20 mm. In the first and the second scan, the respiratory frequency was 15 BPM. In the other scans, the respiratory frequencies were 13, 12, 11, 9, and 7 BPM, respectively. The surrogate respiratory signal was recorded with an air bellows sensor,^(6)^ which was attached to the chest wall platform. All scans were acquired with p=0.081. This pitch is the maximum pitch for a respiratory frequency of approximately 16 BPM. For all respiratory frequencies, the maximum allowed pitch was exceeded.

Each scan was processed in accordance with our clinical routine. First, 10 equidistant phases were reconstructed using a FOV of 500 mm and a slice width of 2 mm. Subsequently, a time‐averaged (AVG) reconstruction and a maximum intensity projection (MIP) reconstruction were created from the 10 phases with TumorLOC (Philips Healthcare, Cleveland, OH). In our clinic, we use these reconstructions for dose calculation and internal target volume (ITV) delineation, respectively.

From the AV G and MIP reconstructions of the dynamic scans, coronal cross sections through the center of the sphere were obtained with PROSOMA 3.3 (MedCom GmbH, Darmstadt, Germany) — our clinical virtual simulation software. Also, superior–inferior radiodensity profiles through the center of the spheres were extracted with IMAGEJ 1.48r (National Institutes of Health, Bethesda, MD). The coronal cross sections from the 6 AVG, as well as the 6 MIP reconstructions, were qualitatively compared.

The measured radiodensity profiles were compared to calculation. For a sphere with radius r (in mm) with a homogenous radiodensity $\rho_{1}$ (in HU) that performs a sinusoidal movement described by Eq. (6) in a homogenous medium with radiodensity $\rho_{0}$, the superior–inferior radiodensity profile through the center of the sphere in the AVG reconstruction can be calculated analytically.

The radiodensity profile can be derived by considering a one‐dimensional segment of length 2r that oscillates along the z‐axis between positions ‐a and +a. From the motion pattern follows the dwell time of the segment at each Z coordinate as a fraction of the oscillation period T. Multiplying the result by the radiodensity of the line segment yields the radiodensity profile.

A little algebra yields a>r:

(7) $$\begin{array}{l} \rho (z)\\ =\{\begin{array}{ll} \rho_{0} & for\left|z\right|>a+r\\ \rho_{0}+\frac{\rho_{1}-\rho_{0}}{2\pi }\left\{\pi -2\arcsin\left(\frac{\left|z\right|-r}{a}\right)\right\} & for\;a-r<\left|z\right|<a+r\\ \rho_{0}+\frac{\rho_{1}-\rho_{0}}{\pi }\left\{\arcsin\left(\frac{\left|z\right|+r}{a}\right)-\arcsin\left(\frac{\left|z\right|-r}{a}\right)\right\} & for\left|z\right|<a-r \end{array} \end{array}$$

where ρ(z) is the radiodensity along the superior–inferior axis. The values of $\rho_{0}$ and $\rho_{1}$ were obtained from the AVG reconstruction of the stationary scan and are the radiodensities of cedar and polystyrene, respectively.

The theoretical MIP reconstruction of a sphere with radius r (in mm) that moves in a sinusoidal pattern described by Eq. (6) represents a spherocylinder. The length (l; in mm) of such a structure through the center along the superior–inferior axis is:

(8) l=2a+2r

In our situation with a=20 mm and r=15 mm, l=70 mm. The full width at half maximum (FWHM) of each measured MIP profile was compared to the theoretical value of 70 mm.

#### B.2 Effect of FOV reduction

From Eq. (1) it might be inferred that a reduction of the reconstructed FOV can be used to improve the image quality if the scan was acquired with a pitch higher than the maximum pitch. When the reconstructed FOV is reduced, the difference between the actual pitch and the maximum allowed pitch will become smaller.

To test this hypothesis, we repeated the phantom measurements (see Materials & Methods section B.1 above) for respiratory frequencies of 12 and 9 BPM. From both scans, AVG and MIP reconstructions were made with a FOV of 500 mm and a FOV of 300 mm. Then, coronal cross sections were obtained in a similar way as in the first experiment. Subsequently, the coronal cross sections obtained with FOV=300 were compared to those obtained with FOV=500 mm.

## III. RESULTS

### A. Nomogram

**Figure 2:**
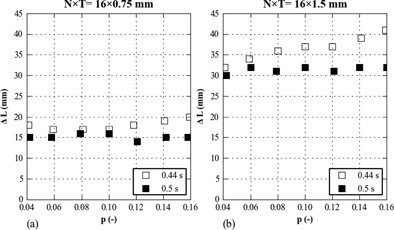
Overranging (ΔL) as function of the pitch (p) for detector collimations (N×T) of (a) 16×0.75 mm and (b) 16×1.5 mm.

Figure 2 shows the results of the overranging measurements. With a detector collimation of 16×0.75 mm, the mean overranging was 18 (16–20) mm and 15 (14–16) mm for a gantry rotation time of 0.44 and 0.5 s, respectively. With a detector collimation of 16×1.5 mm, the mean overranging was 37 (32–41) mm and 31 (30–32) mm for a gantry rotation time of 0.44 and 0.5 s, respectively. To avoid the introduction of the gantry rotation time in Eq. (5) and to preserve the 2:1 ratio of the two detector collimation settings, ΔL was set to 20 mm and 40 mm for a detector collimation of 16×0.75 and 16×1.5 mm, respectively.

**Figure 3:**
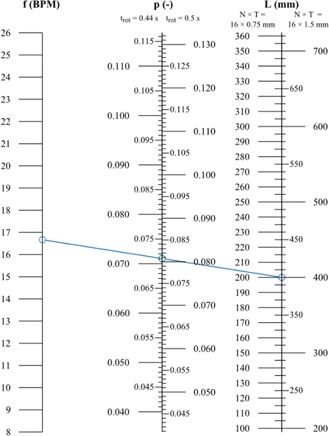
Nomogram for a field of view of 500 mm $\left(f=\text{respiratory}\;\text{frequency},\;\text{BPM}=\text{breath}\;\text{per}\;\text{minute},\;{p=\text{pitch},\;t}_{\text{rot}}=\text{gantry}\;\text{rotation}\;\text{time},\;L=\text{scan}\;\text{length},\;N\times T=\text{detector}\;\text{collimation}\right)$. A blue line has been added to illustrate how to use the nomogram.

Figure 3 shows the nomogram. Respiratory frequencies of 8 to 26 ${\text{min}}^{-1}$ are horizontally linked to their maximum pitches and maximum scan lengths. The minimum pitch that can be set is 0.04. In case of a field of view of 500 mm, this means that the patient's respiratory frequency must at least be 7.8 and 8.9 BPM for a gantry rotation time of 0.44 and 0.5 s, respectively.

### B. Phantom studies

#### B.1 Effect of $p>p_{\text{max}}$

**Figure 4:**
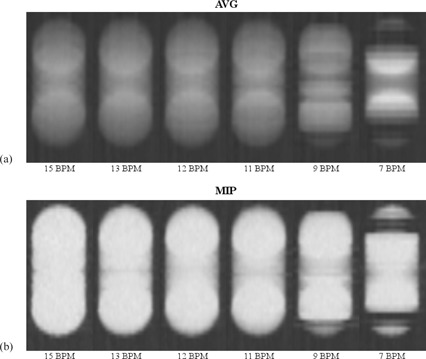
Coronal cross sections from the first phantom study. In (a), the cross sections from the time‐averaged (AVG) reconstructions are shown for different respiratory frequencies (BPM). In (b), those of the maximum intensity projection (MIP) reconstructions are shown. All images were captured with our clinical window level of −402 HU and window width of −987 HU.

**Figure 5:**
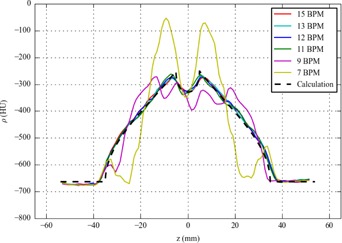
Time‐averaged (AVG) radiodensity profiles from the first phantom study (ρ=radiodensity, z=superior–inferior position, BPM=breath per minute). The calculated profile was created using Eq. (7) with (ρ=radiodensity, z=superior–inferior position, BPM=breath per minute).

As can be seen in Fig. 4, the coronal cross sections for 15, 13, 12, and 11 BPM show strong similarity. For 9 and 7 BPM, the cross sections contain motion artifacts. The AV G profiles for 15, 13, 12, and 11 BPM show strong similarity with the calculated profile (see Fig. 5). For 9 and 7 BPM, the AVG profiles clearly differ from the calculated profile.

The FWHMs of the MIP profiles for 15, 13, 12, and 11 BPM were in the range of 68–70 mm. They agree with the theoretical value of 70 mm, considering the measurement uncertainty of 2 mm(=1 slice width). For 9 and 7 BPM, the MIP profiles did not resemble a step function due to motion artifacts. Their FWHMs were therefore not determined.

### B.2 Effect of FOV reduction

**Figure 6:**
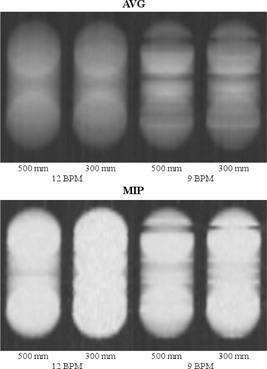
Coronal cross sections from the second phantom study. In (a), the cross sections from the time‐averaged (AVG) reconstructions are shown for different respiratory frequencies (BPM). In (b), those of the maximum intensity projection (MIP) reconstructions are shown. All images were captured with our clinical window level of −402 HU and window width of −987 HU.

The results of the second experiment are shown in Fig. 6. The FOV reduction from 500 to 300 mm did not lead to a clear improvement of the AVG and MIP reconstructions. For 9 BPM, the two reconstructions for a FOV of 300 mm still contain motion artifacts.

## IV. DISCUSSION

The nomogram (see Fig. 3) can be used to obtain a suitable pitch. This pitch is both low enough to illuminate each voxel of patient anatomy for an entire respiratory cycle and high enough to complete the scan within 120 s. First, draw a line from the patient's respiratory frequency prior to the scan (f on the left) to the desired scan length (L on the right). Then, read off the pitch (p in the center) at the intersection. Make sure to use the correct scales. As an example, a solid line (blue) has been added to the nomogram. This line connects an average respiratory frequency^(7)^ of 16.7 BPM to a scan length of 400 mm 400 mm(N×T=16×1.5 mm). The intersection with the central axis indicates pitches of $0.071(t_{\text{rot}}=0.44\;s)$ and $0.081(t_{\text{rot}}=0.5\;s)$.

By construction — the respiratory frequencies are horizontally linked to maximum pitches and maximum scan lengths — only declining or horizontal lines yield pitches that are allowed. Inclining lines will yield pitches that exceed the maximum pitch.

Selecting the maximum pitch (horizontal line in the nomogram) is not a good idea, since a drop of the respiratory frequency during the scan would result in motion artifacts.

When the patient's respiratory frequency prior to the acquisition is too low with respect to the desired scan length (inclining line in the nomogram), (s)he can be coached to breath faster in order to obtain a declining line. Alternatively, the scan length can be reviewed and shortened, if possible. Another solution can be to increase the detector collimation to 16×1.5 mm when it is 16×0.75 mm. However, the detector collimation of 16×0.75 mm might be of limited use. For typical respiratory frequencies of 10 to 20 BPM, the maximum scan lengths associated with this detector collimation are 127 to 274 mm. This is considerably less than the typical scan length of 400 mm for our patients.

Although the nomogram could be used to set individualized pitches, it is more practical to define a default pitch in a thoracic 4D CT scanning protocol (e.g., to prevent the risk of accidental errors that can arise from manual input). Our requirement is that the default pitch enables us to define scan lengths up to 425 mm. To cover 425 mm(N×T=16×1.5 mm) within $120\;s,\;p={0.081(t}_{\text{rot}}=0.5\;s)$ or $p={0.071(t}_{\text{rot}}=0.44\;s)$ is required. These values can be read off from the nomogram by drawing a horizontal line from L=425 mm(N×T=16×1.5 mm) to the p‐scale. Since our clinical scan protocols all use $t_{\text{rot}}=0.5\;s$, we decided on a default pitch of 0.081. This is the maximum pitch for a respiratory frequency of approximately 16 BPM. For lower frequencies, our default pitch exceeds the maximum pitch. Theoretically, this can cause motion artifacts.

The first experiment showed no relevant loss of image quality in AVG and MIP reconstructions for respiratory frequencies down to 10 BPM, which suggests that Eq. (1) yields an underestimation of the maximum pitch. Moreover, in the second experiment, a FOV reduction did not lead to a clear improvement of the image quality in AV G and MIP reconstructions. It is noteworthy that, if the FOV term in Eq. (1) is omitted, the minimum respiratory frequency associated with our default pitch is actually 10 BPM.

Our observations may not be applicable to clinical routines in which the separate phases are used. In the AVG and MIP reconstructions, possible artifacts in the separate phases may have compensated each other.

The results were obtained under well‐controlled and ideal conditions. The regular sinusoidal motion pattern was directly linked to the surrogate respiratory signal. In clinical practice, the largest error source in 4D CT imaging is irregular patient breathing.^(8)^ The amplitude of the phantom motion (4 cm peak to peak) was worst‐case;^(8)^ smaller amplitudes are expected to cause less motion artifacts.

We have instructed our radiation therapy technologists (RTTs) to make sure that the patient's respiratory frequency is at least 12 BPM prior to the scan. If the frequency is lower, the RTTs coach the patient to breathe faster or there is discussion with the radiation oncologist as to whether the scan length can be shortened.

## V. CONCLUSIONS

We presented a nomogram that can be used to select a suitable helical 4D CT pitch for patients with thoracic malignancies whose planning study is made with a Brilliance CT Big Bore. This pitch is both low enough to illuminate each voxel of patient anatomy for an entire respiratory cycle and high enough to complete the scan within 120 s.

We also assessed the effects on the image quality of a pitch that exceeds the maximum pitch, and of a FOV reduction. It was shown that, for AVG and MIP reconstructions, the manufacturer's maximum pitch equation yields an underestimation due to its FOV term.
